# Carbon ion irradiation of the human prostate cancer cell line PC3: A whole genome microarray study

**DOI:** 10.3892/ijo.2014.2287

**Published:** 2014-02-03

**Authors:** ANNELIES SUETENS, MARJAN MOREELS, ROEL QUINTENS, SABINA CHIRIOTTI, KEVIN TABURY, ARLETTE MICHAUX, VINCENT GRÉGOIRE, SARAH BAATOUT

**Affiliations:** 1 Radiobiology Unit, Molecular and Cellular Biology, Belgian Nuclear Research Centre (SCK•CEN), Mol;; 2 Radiation Protection, Dosimetry and Calibration Expert Group, SCK•CEN, Mol;; 3 Department of Radiation Oncology and Center for Molecular Imaging, Radiotherapy and Oncology, Institute of Experimental and Clinical Research (IREC), Université Catholique de Louvain (UCL), Brussels;; 4 Department of Molecular Biotechnology, Ghent University, Ghent, Belgium

**Keywords:** microarray, carbon ion therapy, motility genes, biomarkers, PC3 prostate adenocarcinoma

## Abstract

Hadrontherapy is a form of external radiation therapy, which uses beams of charged particles such as carbon ions. Compared to conventional radiotherapy with photons, the main advantage of carbon ion therapy is the precise dose localization along with an increased biological effectiveness. The first results obtained from prostate cancer patients treated with carbon ion therapy showed good local tumor control and survival rates. In view of this advanced treatment modality we investigated the effects of irradiation with different beam qualities on gene expression changes in the PC3 prostate adenocarcinoma cell line. For this purpose, PC3 cells were irradiated with various doses (0.0, 0.5 and 2.0 Gy) of carbon ions (LET=33.7 keV/*μ*m) at the beam of the Grand Accélérateur National d’Ions Lourds (Caen, France). Comparative experiments with X-rays were performed at the Belgian Nuclear Research Centre. Genome-wide gene expression was analyzed using microarrays. Our results show a downregulation in many genes involved in cell cycle and cell organization processes after 2.0 Gy irradiation. This effect was more pronounced after carbon ion irradiation compared with X-rays. Furthermore, we found a significant downregulation of many genes related to cell motility. Several of these changes were confirmed using qPCR. In addition, recurrence-free survival analysis of prostate cancer patients based on one of these motility genes (*FN1*) revealed that patients with low expression levels had a prolonged recurrence-free survival time, indicating that this gene may be a potential prognostic biomarker for prostate cancer. Understanding how different radiation qualities affect the cellular behavior of prostate cancer cells is important to improve the clinical outcome of cancer radiation therapy.

## Introduction

Recent advances in radiotherapy, such as hadrontherapy, have been added as a radiation treatment choice for specific types of cancer. The inverted depth-dose profile and the sharp dose fall-off after the Bragg peak offered by charged particle beams allow for a more precise localization of the radiation dosage to the tumor (1). Due to this greater precision the surrounding healthy tissue receives a much lower dose compared to conventional radiotherapy with photons. The use of carbon ion beams offers, besides this ballistic advantage, also a biological advantage. High-linear energy transfer (LET) carbon ion radiation has been shown to have a higher relative biological effectiveness (RBE) compared to conventional low-LET photon therapy (2), and is therefore more effective in inducing DNA damage, cell cycle arrest and cell death in tumor cells (3–5). This accounts for the highly lethal effects of carbon ions, even on radioresistant (with respect to X-rays) tumors. Currently, carbon ion radiotherapy has been approved for treatment of specific types of cancer, including prostate cancer (6–8).

Prostate cancer is the second most frequently diagnosed cancer and the sixth leading cause of cancer mortality in males worldwide (9). Most prostate cancer-related deaths are due to metastasis (9–11). So far, first results obtained from prostate cancer patients treated with carbon ion therapy, showed good local control and high local and biochemical relapse-free rates (8,12–15). However, an increased survival rate may be accompanied by potential long-term biological consequences after carbon ion radiotherapy, including metastasis (16,17).

Metastases occur when cancerous cells acquire properties which allow them to detach from the original cancer site and adhere to a target organ to form a new tumor (10). Changes in gene expression can dysregulate cell signaling pathways, thereby leading to changes in functional cell behavior which can ultimately result in cancer metastasis (18,19). Acquiring these properties is a multi-step process starting with tumor growth, followed by detachment of cancer cells, migration and invasion in the surrounding tissue, circulation into blood vessels and implantation to a distant organ (20).

Therapeutic intervention may augment the metastatic potential of cancer cells, and many authors have suggested that a sub-lethal dose of photon irradiation promotes cancer cell metastasis by increasing their migration and invasion potential (21–27). In particular, Zhou *et al* (22) irradiated cancer cell lines from different organ sites and showed that γ-irradiation increased the capacity for migration and invasion, a finding that was also seen in glioblastoma cells (21). Interestingly, previous *in vitro* studies which compared the effects of particle and photon beams indicated that particle beams can decrease the migration potential of cancer cells whereas in most cases X-irradiated samples showed only a slight decrease or even an increase in their migration potential (28–32). Ogata *et al* (30) irradiated human fibrosarcoma cells with X-rays, protons or carbon ion beams and observed a dose-dependent decrease in cell migration and invasion caused by particle irradiation, whereas low doses of X-rays facilitated the process. Goetze *et al* (28) irradiated glioblastoma cells and colorectal carcinoma cells with carbon ions or X-rays and found that carbon ion irradiation suppressed the migration potential in both cell lines, while X-rays suppressed the migration potential only in the colon carcinoma cells, indicating a cell type-specific effect.

The fate of a cancer cell after radiotherapeutic intervention is believed to be controlled by a network of signaling pathways that lead to different modes of cell death or survival (33). Several studies have compared changes in gene expression of cancer cells induced by particle and photon beams (29,34–37). Particle beams were found to induce more changes in the number of genes that were differently expressed, as well as the magnitude of (dose-dependent) gene expression changes. Pathways in which these genes were involved were mostly related to cell cycle regulation, invasion and angiogenesis which may be associated with an enhanced aggressive phenotype of the cancer cells. To our knowledge, the effect of carbon ion beam radiation on gene expression of prostate cancer cells *in vitro* has not been verified so far.

The main aim of this study was to investigate the impact of carbon and X-irradiation on gene expression levels of the prostate adenocarcinoma cell line PC3 using whole-genome microarrays. This highly invasive cell line exhibits strong metastatic activity (38) and is widely used as an *in vitro* model to investigate the biological and cellular responses of human prostate cancer cells. Our results demonstrate that carbon ion irradiation induced stronger effects at the level of gene expression compared to similar doses of X-rays. Specifically after carbon irradiation, a more pronounced, dose-dependent down-regulation of genes involved in cell migration and motility was observed.

## Materials and methods

### Cell culture

Human PC3 prostate adenocarcinoma cells were obtained from the American Type Culture Collection (ATCC, Molsheim, France). They were cultured in F-12K medium (ATCC) supplemented with 10% fetal bovine serum (FBS) (Gibco, Life Technologies, Ghent, Belgium). Cell cultures were maintained in a humidified incubator (37°C; 5% CO_2_). For all irradiation experiments the same passage number of cells was used. For all conditions, we used four separate replicates.

### X-irradiation

Cells were plated at a density of 3.5×10^5^ cells in 12.5 cm^2^-tissue culture flasks (Falcon; VWR, Leuven, Belgium). After seeding, medium was replaced and cells were irradiated in a horizontal position with different doses of X-rays (0.0, 0.5 and 2.0 Gy) (Pantak HF420 RX machine; 250 kV, 15 mA, 1.2 mm Al equivalent and 1 mm Cu-filtered X-rays) and a calculated dose rate of 0.25 Gy/min. After irradiation, cells were further incubated for 8 h.

### Carbon ion irradiation

Cells were plated in 175 cm^2^-tissue culture flasks (Falcon; VWR). Cells were transported by car in a transportable incubator at 37°C to the Grand Accélérateur National d’Ions Lourds (GANIL) (Caen, France). During cell transportation, culture flasks were completely filled with medium. After arrival, medium was replaced and cells were placed overnight in a humidified incubator. The following day 3.5×10^5^ cells were plated in 12.5 cm^2^-tissue culture flasks (Falcon; VWR). After seeding, the flasks were completely filled with medium to allow irradiation in a vertical position. Sub-confluent cells were irradiated with a ^13^C beam with an initial energy of 75 MeV/u and a flux of 6.24×10^5^ cm^−2^sec^−1^ at the D1 beam line at GANIL facility. The dosimetry was performed by physicists of CIMAP group at GANIL. It is based on the monitoring of the total ion current using the X-ray emission by a metallic thin foil inserted in the beam path. For fluxes lower than 10^6^ cm^−2^ sec^−1^, a calibration factor between these secondary photons and the particle flux is obtained by counting the ion tracks measured on CR39 track detectors. A second step for checking the linearity is performed by a conventional ionization chamber. Taking into account the different layers before the beam arrives at the sample, the resulting LET was 33.7±1.6 keV/*μ*m, calculated with the SRIM version 2011 code (39). To irradiate the samples at 0.0, 0.5 and 2.0 Gy absorbed doses, the requested fluences were 0, 9.3×10^6^ cm^−2^ and 3.7×10^7^ cm^−2^ by using the following equation:

$$\mathrm{Fluence}\;\left(\frac{\mathrm{Particles}}{cm^{2}}\right)=\frac{D\left[\mathrm{Gy}\right]}{1.6\;\;\;*\;\;\;10^{-9}\;\;x\;\;\;\;\mathrm{LET}\;\;\;\left[\mathrm{keV}/\mu m\right]}$$

The spot size of the beam was around 4×4 mm^2^. Immediately after irradiation, medium in the flasks was removed (except for 2 ml) in which the cells were further incubated for 8 h. Control samples were treated under similar conditions, including transportation and positioning identical to, and simultaneous with, that of treated samples.

### RNA extraction

Medium was removed from the flasks 8 h after irradiation, cells were rinsed with phosphate-buffered saline (Gibco) and finally collected in 350 *μ*l RLT buffer (Qiagen, Venlo, The Netherlands) with β-mercapto-ethanol (Sigma-Aldrich, Bornem, Belgium). Total RNA was isolated according to the manufacturer’s instructions using AllPrep DNA/RNA/protein mini kit (Qiagen). The quantity of RNA was measured with the NanoDrop Spectrophotometer (NanoDrop Products, Wilmington, DE, USA) and the quality was assessed with an Agilent Bioanalyzer (Agilent Technologies, Diegem, Belgium). RNA was stored at −80°C until further processing.

### Microarrays and data analysis

Microarrays were prepared as reported previously by El-Saghire *et al* (40). After labeling, samples were hybridized onto Human Gene 1.0 ST Array Chips (Affymetrix, High Wycombe, UK). Raw data (.cel-files) were then imported into Partek software (Partek Genomic Suite v5.) (Partek Inc., St. Louis, MO, USA). Quality control was performed according to Partek software instructions. Based on the PCA of all 24 samples three samples were indicated as outliers and removed from the analysis. To test for differential expression two-way ANOVA analysis (with experimental conditions and dose as factors) was performed. P-values were adjusted for multiple corrections using false discovery rate (FDR) as described by the Benjamini and Hochberg (41) procedure. Genes were considered as being differentially expressed (DEX) when the fold-change (FC) was 2≤FC≤−2 and FDR ≤0.05. DEX genes were examined for functional enrichment using the ToppFun tool (http://toppgene.cchmc.org/) and applying an FDR corrected P-value of 0.05 for statistical significance. Gene Ontology lists of Molecular Function and Biological Processes were examined with the visualization tool AmiGO (v1.8) available on the Gene Ontology website. Further examination of the data set was performed with the online tool CateGOrizer in order to get a more precise view of the most important affected processes (42). Finally, DEX genes were filtered based on the cell motility gene set found on the Gene Ontology website (Table I).

### Functional enrichment analysis

In order to determine to which extent specific gene sets were influenced by changes found in genes with Partek software, a gene set enrichment analysis (GSEA) was performed. GSEA allows calculating statistically significant enrichment of a set of DEX genes towards specific pathways or biological processes. Gene ontology databases for molecular functions, biological processes and cellular components were analyzed separately. Analysis was performed using GSEA software (v2.0.10) (43,44). Number of permutations was set at 1,000; gene sets were set as permutation type and minimum gene set size was 10. Otherwise, default settings were maintained. Gene sets were considered to be enriched with a FDR P-value ≤0.05. Enrichment maps, which indicate relationships between the enriched sets, were visualized for gene sets coding for cellular components using the Enrichment Map plug-in for the Cytoscape network visualization software (45).

### cDNA synthesis

cDNA was synthesized with a GoScript™ Reverse Transcription System (Promega, Leiden, The Netherlands) on a GeneAmp PCR System 2700 (Applied Biosystems, Foster City, CA, USA). We used 0.4 *μ*g RNA in 20 *μ*l reactions as per the manufacturer’s instructions. cDNA samples were stored at −20°C until further reverse transcriptase PCR analysis.

### RT-qPCR

Primers for target gene expression analysis (Table II) were purchased as pre-made assays (TaqMan Gene Expression Assay) (Applied Biosystems). Assays were performed according to the manufacturer’s instructions. Briefly, 2 *μ*l cDNA was added to 1 *μ*l TaqMan Gene expression Primer, 10 *μ*l TaqMan^®^ Fast Advanced Master Mix and 7 *μ*l RNase free water. Assays were run on a 7500 Fast Real-Time PCR system (Applied Biosystems). First, efficiency of the primers was tested, using a five-step dilution series of an independent control sample (Table II). Expression ratios (R) were calculated using the method as described by Pfaffl (46). Finally, data were normalized by a log_2_ transformation and data are presented as average log_2_(R) ± SD.

### Statistical analysis of RT-qPCR

Statistics were performed with GraphPad Prism 5.00. Statistical significance of differences between log_2_(R) of control and each experimental condition was determined using one-tailed Mann-Whitney tests. P-values ≤0.05 were considered statistically significant.

### Kaplan-Meier analysis of public patient data

In order to assess the clinical relevance of our findings we cross-referenced our results with published patient data. We worked with independent, publicly accessible microarray data of prostate cancer patients (47,48) which could be imported into Partek. Our genes were cross-referenced in the patient data. Data from the study of Taylor *et al* (48) were imported in Partek and transformed with a two-base logarithm. Columns with ‘gene status’ were inserted, status was appointed as ‘high’ (expression data more than mean + SD); ‘intermediate’ (expression data between the mean + SD and mean − SD); and ‘low’ (expression data less than the mean − SD). Data from the study of Gulzar *et al* (47) were imported to Partek. Normal samples and duplicates were removed from the set. Properties, such as status and time to recurrence, were added to the data. Then, the original data were untransformed (anti-log_2_). Columns were filtered based on a list of our genes of interest. Columns of ‘gene status’ were inserted, status being appointed as ‘high’ (expression data >1.3 vs. reference RNA); ‘intermediate’ (expression data ≤1.3 and ≥0.77); and ‘low’ (expression data <0.77 vs. reference RNA). When status was appointed data were transferred to GraphPad Prism 5.00 for Kaplan-Meier analysis based on patient separation in high, intermediate or low gene status. Differences in recurrence-free survival were considered significant at log-rank P≤0.05.

### Ethics statement

All patient data were obtained from Taylor *et al* (48) and Gulzar *et al* (47) after which they were made publicly available. Therefore, their use was not classified as human subjects research and no Institutional Review Board approval was needed.

## Results

Gene expression profiles of PC3 cells were analyzed 8 h after exposure to different doses of carbon ion (LET=33.7 keV/*μ*m) and X-irradiation. Microarray data analysis followed by GO analysis allowed the identification of genes, as well as pathways, which were enriched among statistically significant genes. Finally our data were cross-referenced with observations from other studies based on clinical data (47,48).

### Principal component analysis (PCA)

**Figure 1.**
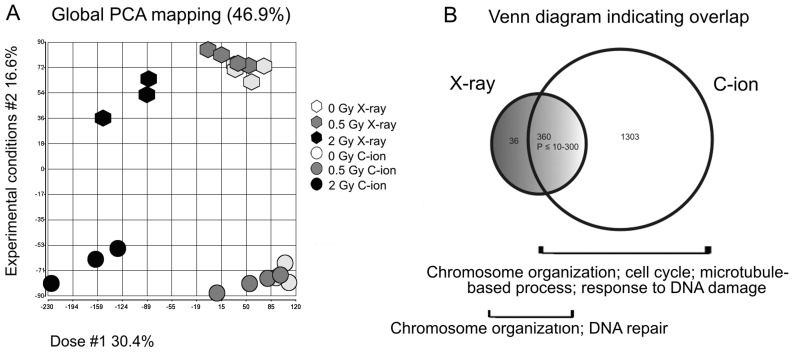
Radiation-induced changes in global gene expression. (A) 2D Principal component analysis (PCA) for PC3 cells after various doses of carbon ion irradiation or X-rays. PCA is based on global gene expression patterns for each irradiation condition. Analysis revealed distinct clustering in four groups of which the percent variability explained by the components dose and experimental conditions was 30.4 and 16.6%, respectively. (B) Venn diagrams showing overlap of significantly altered genes after 2.0 Gy irradiation of either beam quality. After 2.0 Gy carbon ion irradiation (white circle) and 2.0 Gy X-rays (grey circle) there are respectively 1,663 and 396 genes differentially expressed (DEX). There was a very significant overlap in gene expression changes between the conditions.

PCA representing the complete gene expression profile presented in two dimensions is shown in Fig. 1A. Data indicated similarity between samples, whereby shorter distance corresponded with greater similarity. Samples clustered together according to the dose (first component) and experimental conditions (second component), which described respectively 30.4% and 16.6% of the variance within the data when considering all genes. Differences in non-irradiated control samples with respect to experimental conditions were most likely due to transport of the cells to the irradiation facility in France. Low biological variance within control samples and samples irradiated with 0.5 Gy resulted in distinct clusters for these conditions. Samples irradiated with 2.0 Gy were more widely distributed over the plot indicating increased biological variation after irradiation with this dose. PCA also demonstrated a distinct shift in gene expression after 2.0 vs. 0.0 Gy for both carbon ion and X-irradiation, while only a small shift was observed after 0.5 Gy of either beam type.

### Differential gene expression after exposure to carbon ion irradiation and X-rays

Carbon ion irradiation induced very profound effects on gene expression levels. Therefore, very strict criteria (−2≥ FC ≥2; FDR ≤0.05) were used in our analysis for considering genes as being DEX. We found that 8 h after exposure to 2.0 Gy carbon ion irradiation the expression of 1,663 genes was changed at least two-fold (Table III). Of these, 69% were downregulated and 31% were upregulated. In contrast, after 2.0 Gy X-radiation only 396 genes were significantly changed. Similar to carbon ion irradiation, the majority of the DEX genes after X-ray exposure were downregulated (79%). Out of these 396 genes, 360 genes were also DEX after exposure to carbon ion irradiation which shows that there was a very significant (P≤10^−300^) overlap in the molecular response of PC3 cells to similar doses of high- and low-LET radiation (Fig. 1B). Under these strict statistical conditions, no genes were significantly changed after exposure to 0.5 Gy of either radiation type.

**Figure 2.**
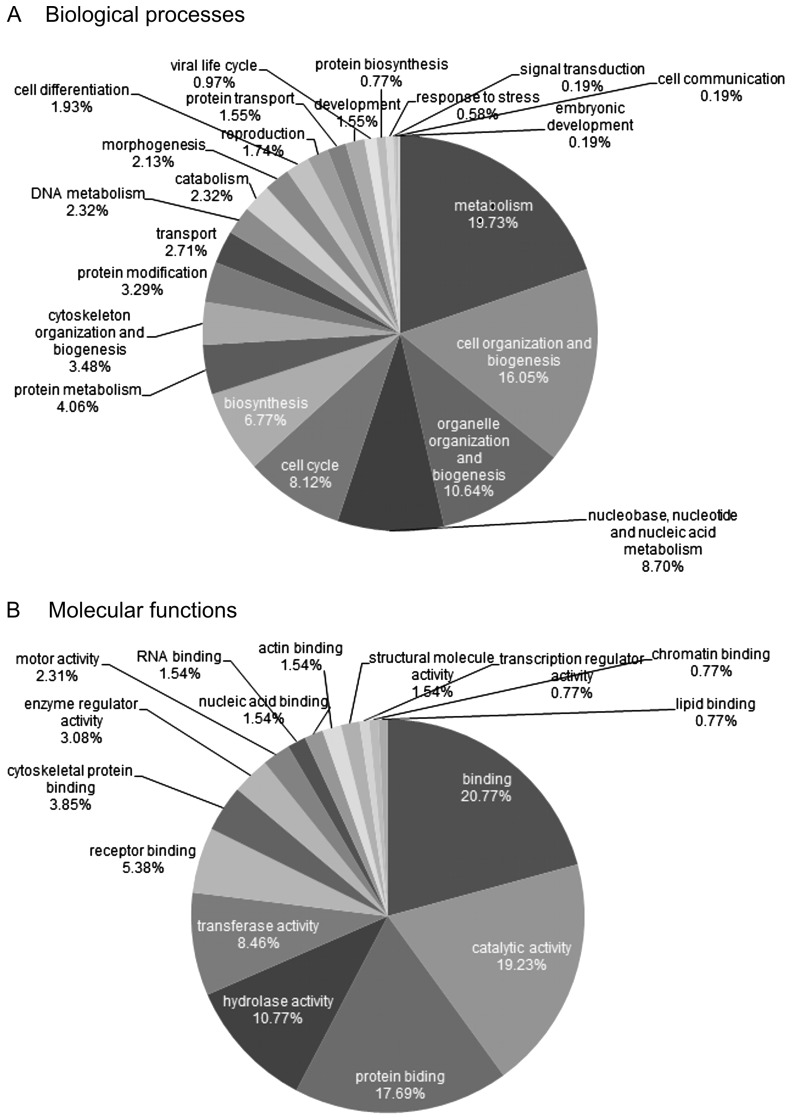
Pie chart of the most important (A) biological processes and (B) molecular functions which are affected by 2.0 Gy carbon ion irradiation.

Functional enrichment of the DEX genes after 2.0 Gy carbon ion irradiation was examined using the ToppFun tool (data not shown). In addition, in order to get a better idea of the categories to which the gene sets belong to, the full gene set was slimmed with CateGOrizer. For the enriched biological processes CateGOrizer identified 21 classes (Fig. 2A), of which the majority (19.73%) was involved in cell metabolism. Next to this, a high percentage was involved in cell (16.05%) and organelle organization (10.64%). In addition, 8.12% of the gene sets were involved in cell cycle related processes. For the affected molecular functions 16 categories were identified (Fig. 2B). Many binding processes were affected (20.77% general binding; 17.69% protein binding; 5.38% receptor binding; 3.85% cytoskeletal protein binding; 1.54% RNA binding; 1.54% nucleic acid binding; 1.54% actin binding; 0.77% chromatin binding and 0.77% lipid binding). Also various enzymatic activities were influenced by radiation (19.23% catalytic; 10.77% hydrolase; 8.46% transferase activity and 3.08% enzyme regulator activity).

### Functional enrichment analysis

For GSEA, 376, 845 and 202 gene sets, related to molecular functions (c5.mf.v3.1), biological processes (c5bp.v3.1) and cellular components (c5.cc.v3.1) respectively, were examined for enrichment among the DEX genes. In general, gene sets were mostly enriched (upregulated) in the control samples, corresponding with the vast amount of genes which are downregulated after irradiation. After 0.5 Gy carbon ion irradiation, 16 gene sets related to molecular functions were enriched in the control samples, while only one set was enriched in irradiated samples. After 2.0 Gy carbon ion radiation 57 sets were enriched in the controls. For 0.5 Gy X-irradiation no gene sets were found to be enriched whereas samples irradiated with 2.0 Gy X-rays had 93 enriched sets in control samples compared to three enriched gene sets in the irradiated samples. Gene sets involved in biological processes (data not shown) were only found to be enriched in the control samples. After 2.0 Gy X-irradiation 100 enriched gene sets were found. After carbon ion irradiation, 31 and 62 gene sets were found to be enriched after 0.5 and 2.0 Gy, respectively. Finally, 13 gene sets coding for cellular components (data not shown) were enriched in samples irradiated with 2.0 Gy carbon ion irradiation, whereas for X-rays 11 gene sets were enriched after irradiation with 2.0 Gy X-rays. Most of these gene sets related to mitochondrial structures. In the control samples 17 sets were enriched after 0.5 Gy carbon ion irradiation. For the X-irradiated samples four sets were enriched after 0.5 Gy, while 42 were enriched after 2.0 Gy.

**Figure 3.**
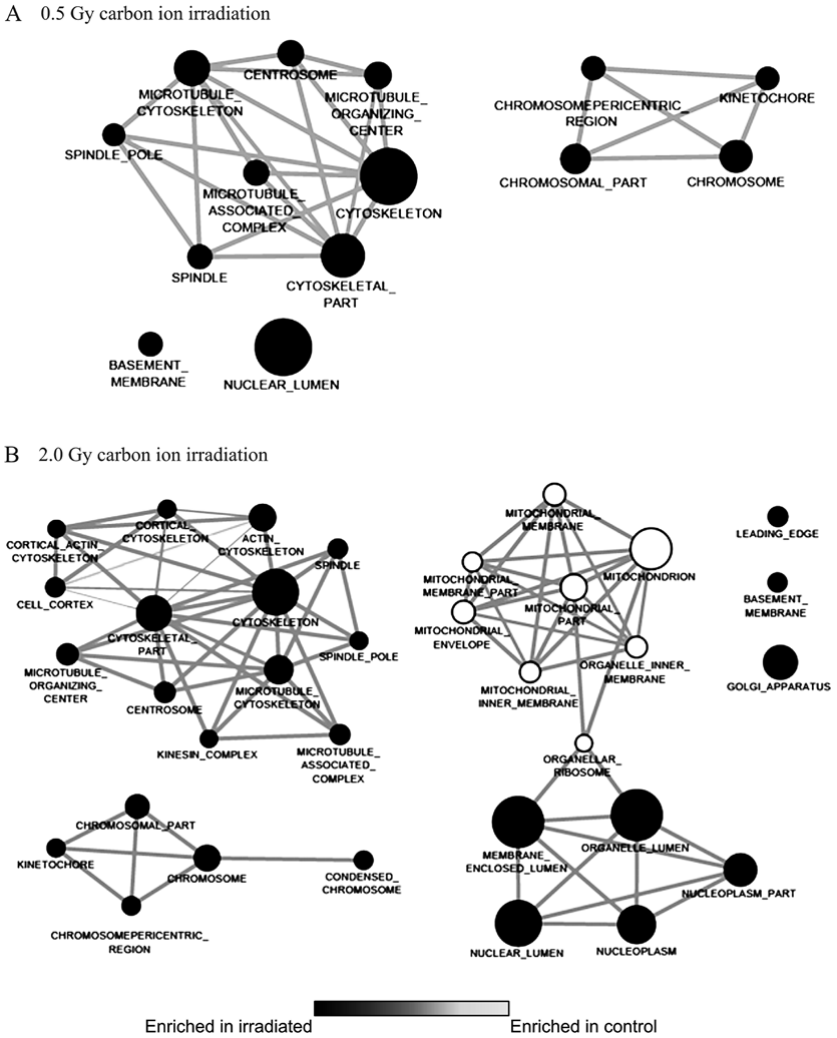
Enrichment maps based on gene set enrichment analysis (GSEA) for GO cellular components after carbon ion irradiation (A, 0.5 Gy, and B, 2.0 Gy). White nodes represent gene sets enriched in irradiated samples (i.e. upregulated in irradiated samples), black nodes represent gene sets enriched in control samples (i.e. downregulated in irradiated samples). Node size correlates with the number of genes within each gene set. Edge width represents the overlap of member genes between gene sets. Enriched gene sets included have a P<0.001 and a false discovery rate (FDR) value P<0.05.

**Figure 4.**
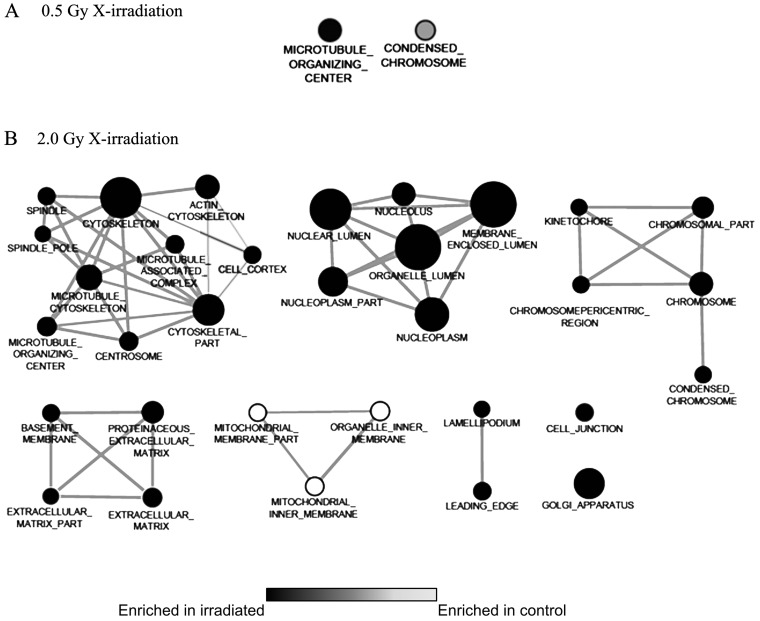
Enrichment Maps based on gene set enrichment analysis (GSEA) for GO cellular components after X-irradiation (A, 0.5 Gy, and B, 2.0 Gy). White nodes represent gene sets enriched in irradiated samples (i.e. upregulated in irradiated samples), black nodes represent gene sets in control samples (i.e. downregulated in irradiated samples). Node size correlates with the number of genes within each gene set. Edge width represents the overlap of member genes between gene sets. Enriched gene sets included have a P<0.001 and a false discovery rate (FDR) value <0.05.

In order to get a more comprehensive view of the enriched gene sets in different conditions, enrichment maps for GO_cellular_components were created with cytoscape (Figs. 3 and 4). The figures show that after 0.5 Gy X-irradiation no related gene sets coding for cellular components were found (Fig. 4). After 0.5 Gy carbon ion irradiation two clusters were identified, which were associated with cytoskeleton and chromosomal components. After 2.0 Gy carbon ion or X-irradiation a cluster associated with chromosomal components were identified, while the cytoskeleton-associated cluster included more gene sets compared to 0.5 Gy irradiation, indicating a dose-dependent effect. Additionally, after 2.0 Gy of either beam quality two clusters associated with nucleus and mitochondrial components were found as well. Finally for the 2.0 Gy X-irradiation also a cluster associated with extracellular matrix were identified.

### Genes involved in cell motility

**Figure 5.**
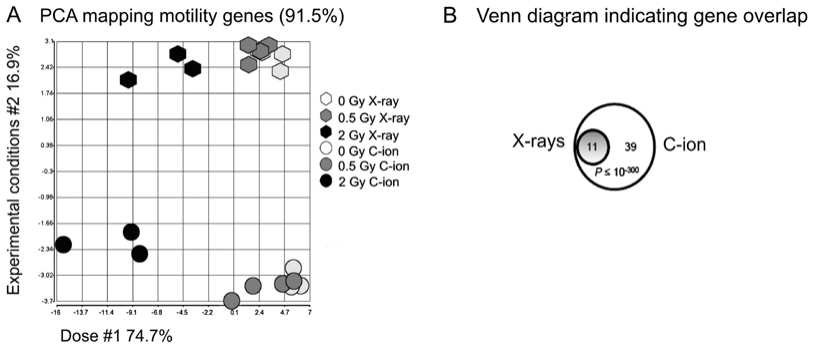
Radiation-induced changes in motility related gene expression. (A) Principal component analysis (PCA) of motility genes revealed distinct clusters similar to global PCA analysis. The percent variability is ∼91.5% explained by the components dose (74.7%) and experimental conditions (16.9%). (B) Venn diagram showing the overlap of motility genes differentially expressed after 2.0 Gy irradiation. All genes found to be differentially expressed by 2.0 Gy X-rays were also regulated by 2.0 Gy carbon ion irradiation.

Given the lack of knowledge of potential long-term effects of carbon ion beams, we were interested in the radiation-induced response of genes related to cell migration. Therefore, we filtered the full gene set based on a list from the Gene Ontology website (GO0048870 - cell motility) containing 715 cell motility related genes. PCA revealed a similar spreading of the clusters (Fig. 5A) compared to the entire transcriptome (Fig. 1A). However, in this case a much larger proportion of the total variance could be explained by the dose (74.7%), indicating that the radiation dose had a very profound effect on the general expression of these genes.

**Figure 6.**
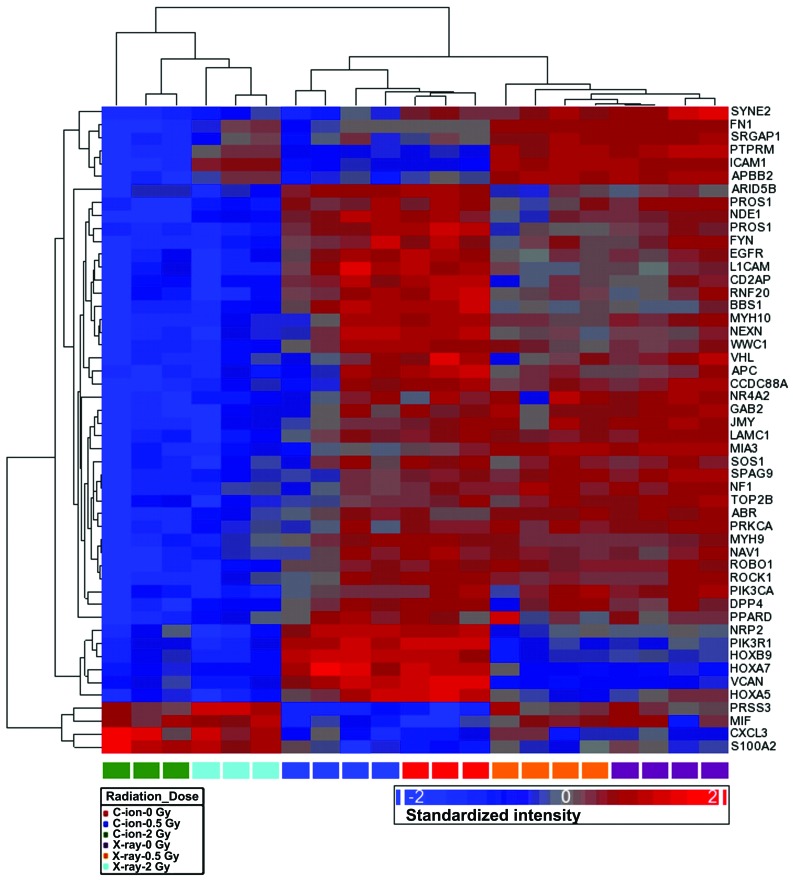
Heat map of the hierarchical clustering for the 50 motility genes which were significantly regulated by 2.0 Gy carbon ion irradiation. First hierarchical separation distinguished samples irradiated with 2.0 Gy on the left of the heat map. Out of the remaining samples, two groups can be distinguished based on beam quality [carbon ions (middle) vs. X-ray (right)].

Out of these 746 genes, 50 genes were found to be DEX (−2≥ FC ≥2; FDR ≤0.05) after 2.0 Gy carbon ion radiation (46 downregulated and 4 upregulated), while only 15 genes were changed (all downregulated) after 2.0 Gy X-irradiation (Table IV). All of the latter were also significantly regulated by 2.0 Gy carbon ion irradiation (Fig. 5B). Unsupervised hierarchical clustering of the 50 motility genes that were DEX after 2.0 Gy carbon ion irradiation is shown in Fig. 6. A clear separation was visible between samples irradiated with 2.0 Gy (of either beam quality) and non-irradiated or low dose (0.5 Gy) irradiated samples. Within this last group two other distinct clusters were visible, separating X-ray and carbon ion samples.

Six out of these 50 motility genes (*NEXN*, *CCDC88A*, *FN1*, *MYH9*, *MYH10* and *ROCK1*) showed at least a four-fold decrease in gene expression after 2.0 Gy carbon ion radiation (Table V). Gene expression levels of these six genes were found to be two to four times more decreased after 2.0 Gy carbon ion radiation compared with 2.0 Gy X-rays. Gene expression changes after 0.5 Gy of both radiation types were not significantly altered, although a dose-dependent trend in expression levels can be seen for both radiation types.

### RT-qPCR analysis

**Figure 7.**
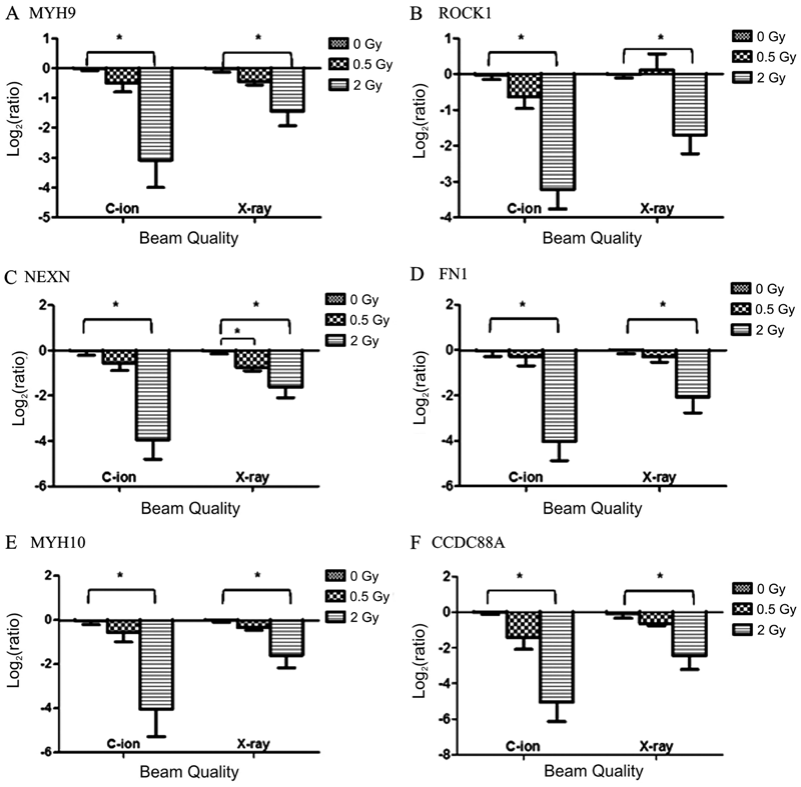
Relative gene expression changes of six selected motility genes 8 h after carbon ion or X-irradiation. Log_2_(ratio) of the expression of (A) *MYH9*, s(B) *ROCK1*, (C) *NEXN*, (D) *FN1*, (E) *MYH10* and (F) *CCDC88A* is presented. Significantly altered gene expression compared to CTRL samples (^*^P≤0.05) based on one-tailed Mann-Whitney tests. RT-qPCR results confirm the downregulation observed by microarray analysis after radiation which was more pronounced after carbon ion radiation when compared to X-rays.

Expression levels of *NEXN*, *CCDC88A*, *FN1*, *MYH9*, *MYH10* and *ROCK1* were validated using quantitative real-time PCR. Average log_2_(R) is presented in Fig. 7. For all but one gene the expression levels tended to decrease after 0.5 Gy (although not significantly). After 2.0 Gy the expression of all six genes significantly decreased. This effect was more visible in carbon ion irradiated samples [log_2_(R) from −3 to −5] compared to X-irradiation [log_2_(R) from −1.5 to −2.5].

### Cross-reference of our results with existing patient data

**Figure 8.**
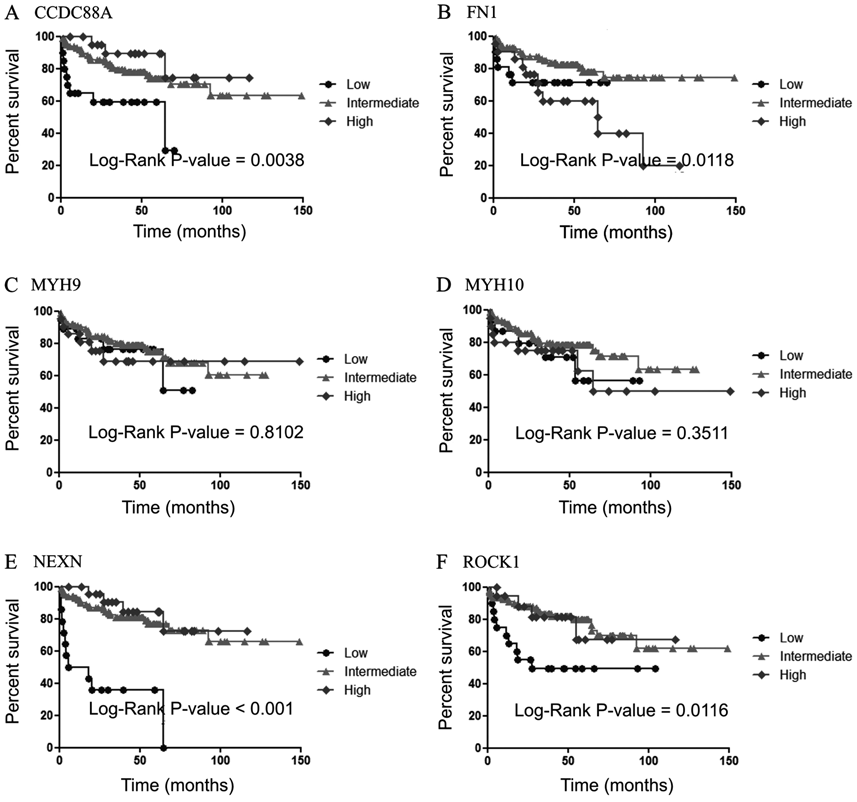
Kaplan-Meier survival analysis of (A) *CCDC88A*, (B) *FN1*, (C) *MYH9*, (D) *MYH10*, (E) *NEXN* and (F) *ROCK1* gene expression performed on the data set of Taylor *et al* (48). Tumor samples were divided into three groups based on whether the gene expression value was high (♦, dark grey); intermediate (▴, light grey); or low (•, black). Differences in survival were found to be significant for *CCDC88A*, *FN1*, *NEXN* and *ROCK1* when log-rank P≤0.05.

**Figure 9.**
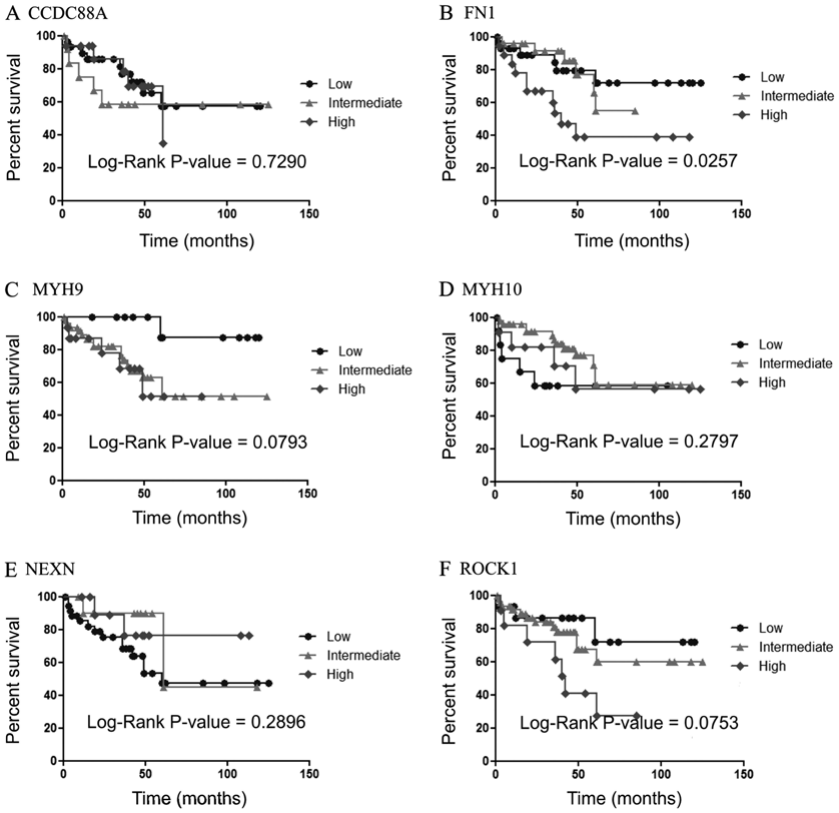
Kaplan-Meier survival analysis of (A) *CCDC88A*, (B) *FN1*, (C) *MYH9*, (D) *MYH10*, (E) *NEXN* and (F) *ROCK1* gene expression performed on the data set of Gulzar *et al* (47). Tumor samples were divided into three groups based on whether the gene expression value was high (♦, dark grey); intermediate (▴, light grey); or low (•, black). Differences in survival were found to be significant for *FN1* (log-rank P=0.0257).

Because of the potential beneficial implications of a down-regulation of motility-related genes for the prognosis of prostate cancer patients, we verified the prognostic power of these genes. We used publicly accessible data sets from two prostate cancer studies (47,48) to generate Kaplan-Meier (recurrence-free) survival curves based on differential gene expression of our six motility genes (Figs. 8 and 9). Within the data set of Taylor *et al* (48) 150 samples were available for analysis for the six genes. Due to missing values in the data set of Gulzar *et al* (47) this data set was limited to 61 samples for *CCDC88A*; 74 for *FN1*; 60 for *NEXN*; 79 for *MYH9*; 77 for *MYH10* and 75 for *ROCK1*. When samples were stratified according to *FN1* expression both data sets correlated with high gene expression and poor prognosis of the patients (log-rank P=0.0118 and 0.0257, respectively). This was the only gene which was concordantly significant in both data sets. High and intermediate levels of *CCDC88A* correlated with a higher overall survival rate (log-rank P=0.0038) within the data set of Taylor *et al*, while analysis of the data set of Gulzar *et al* seemed to show an opposite trend however without statistical significance (log-rank P= 0.7290). High *ROCK1* expression correlated with better survival rates (log-rank P=0.0116) among the patients of Taylor *et al*, whereas within the data set of Gulzar *et al* an opposite trend could be seen. Discrimination based on *NEXN* expression was highly significant within the data set of Taylor *et al*, however in this case low gene expression was associated with poor prognosis (log-rank P≤0.0001). This was not the case within the data set of Gulzar *et al* where no statistical discrimination could be made (log-rank P=0.2896). *MYH9* expression did not show any difference in patient survival in the group of Taylor *et al*. However, within the data set of Gulzar *et al* data appeared to show a trend which correlated intermediate-high expression of *MYH9* with a lower overall survival rate. Therefore, this analysis was performed again by dividing patient samples in two categories, low and intermediate-high. This resulted in a good discrimination between good (low *MYH9* expression) and poor prognosis (log-rank low vs. intermediate P=0.0079; log-rank low vs. intermediate P=0.0073; data not shown). Finally, based on *MYH10* gene expression patient samples could not discriminate between good and poor prognosis in either data set.

## Discussion

Radiation therapy plays an important role in the management of several types of cancer. The fate of an irradiated cancer cell is believed to be controlled by a network of signaling pathways that lead to different modes of cell death or survival. Although most of the cells will die due to the lethal dose, some cells will manage to survive after radiotherapeutic intervention, because they successfully utilize their repair mechanisms. Numerous studies have demonstrated enhanced aggressiveness of surviving cancer cells after conventional radiotherapy, accompanied with an upregulation of genes that favor cell migration, invasion and angiogenesis (21,22,26,49,50). It is well known that carbon ion beams have an increased biolo gical effectiveness compared to X-rays. Therefore, it would not be surprising that both types of radiation can induce different effects at the level of gene expression. So far, the observed differences in gene expression between different radiation qualities are not completely understood. In the present study, we compared the differences in the transcriptional response 8 h after carbon ion (LET=33.7 keV/*μ*m) and X-irradiation of the human PC3 prostate adenocarcinoma cell line. To our knowledge, we are the first to describe global gene expression changes after exposure to different radiation qualities in prostate cancer cells.

### Global radiation-induced gene expression changes

We found that 2.0 Gy carbon ion irradiation induced more pronounced changes in gene expression compared to similar doses of X-rays both in terms of number of genes and magnitude of changes. After carbon ion irradiation four times more genes were altered in PC3 cells. Our results demonstrated that almost 90% of the genes that were significantly altered after 2.0 Gy X-ray overlapped with those that were changed after 2.0 Gy carbon ions, indicating that similar molecular pathways were affected. Furthermore, 69% of the significantly altered genes were downregulated. Under our strict statistical criteria, no significant changes were induced after 0.5 Gy irradiation for both radiation qualities. Our results are in agreement with previous findings of Matsumoto *et al* (34), who examined gene expression in six different melanoma cell lines 1 h and 3 h after exposure to X-rays or carbon ion beams (average dose-LET=50 keV/*μ*m). They found that, after exposure to 2 Gy of both radiation types, several hundreds of genes were differentially expressed at both time-points. Many of these genes showed a greater response to carbon ions compared to X-rays. Similar to our results they found that most of the altered genes were downregulated after radiation exposure. Higo *et al* (37) irradiated three oral squamous cell carcinoma cell lines with X-rays (2, 4 and 8 Gy) and carbon ions (1, 4 and 7 Gy; LET=78 keV/*μ*m). They also found that carbon ion irradiation induced at least a two fold-change in 98 genes for all doses and all cell lines of which 85 genes were upregulated and 13 down-regulated. In agreement with our results, they found more genes altered by carbon ion radiation compared to X-rays (30 genes upregulated and 4 genes downregulated for all doses in all cell lines). However, in contrast with the study by Matsumoto *et al* (34) and our findings most of the genes found in the study of Higo *et al* (37) were upregulated.

All these studies observed a greater impact of carbon beam irradiation on changes in gene expression compared to conventional photon irradiation. However, there are differences in the number of genes that are upregulated or downregulated. Many factors can be responsible for this including the chosen time-point for sample collection. Furthermore, differences in cell type, as well as, physical aspects including irradiation conditions and LET of the beam may have an impact on radiation-induced genome changes.

One limitation of this study is that our results only compare equal doses. We are aware that, considering the higher RBE of carbon ion beams for a similar dose, carbon beams may induce more changes at the gene level compared to X-rays. However, because of limited beam time, only a small number of experiments could be performed and gene expression experiments were selected as first priority over an estimation of the RBE. We therefore analyzed radiation-induced changes in gene expression after equal doses. In view of time-dependent fluctuations (29) in gene expression induced after irradiation and the difficulty of correlating RBE to these time-dependent changes, comparing equal doses for gene expression profiling was considered as a suitable alternative. Indeed Matsumoto *et al* (34) determined RBE for their cell lines and beam qualities but still compared equal doses at different time-points. However, future experiments should include clonogenic survival assay of PC3 cells exposed to carbon ions and X-rays to determine the RBE. Additionally, experiments including more doses and more time-points would greatly contribute to the characterization of differences in carbon ion and X-irradiation.

### Radiation-induced signaling pathways

Enrichment analysis of the DEX genes indicated that the cell cycle, cellular organization and metabolism are affected by 2.0 Gy carbon ion irradiation. Interestingly, although differential expression after 0.5 Gy was not found to be significant for single genes, GSEA did reveal significant changes.

Also other studies have indicated similar pathways being affected by high-LET radiation. Higo *et al* (37) performed Ingenuity Pathway Analysis which indicated significant alteration of several pathways, amongst which the cell cycle, cell movement and cell assembly and organization in carbon irradiated squamous cell carcinoma. Also Matsumoto *et al* (34) observed many changes in the cell cycle-related genes after carbon ion irradiation. In addition, they found that, in four out of six cell lines, upregulated genes were mostly p53 target genes. In our PC3 cell line, which has a mutated p53 status (51), we did not observe many significantly upregulated genes involved in the p53 pathway. Matsumoto *et al* (34) made similar observations with two of their melanoma cell lines, one of which being wild-type, and another having a mutated p53 status. Based on their findings, they suggested that downregulation of gene expression plays a key role in the extra effect of carbon ions compared with X-rays (i.e. an increased radiobiological effectiveness), whereas upregulation of genes relates to sensitivity to both beam qualities (i.e. inherent radiosensitivity of the cell to radiation).

### Cell motility-related gene expression changes

An interesting phenomenon observed in *in vitro* studies is the effect of radiation on cell motility, migration and invasion. Previous studies on low-LET photon irradiation already reported an elevated migration potential of cancerous cells (21,22,26,49,50). So far, however, most studies comparing X-irradiation with particle beams indicated that particle radiation attenuates or even decreases the migration potential of most cancer cell lines (28–32). Therefore, in this study, we more specifically focused our gene expression analysis on genes involved in cell migration and motility. We observed a dose-dependent downregulation after carbon ion irradiation in several genes involved in these pathways, which was significant after 2.0 Gy radiation. Again, the number and amplitude of gene expression changes after X-irradiation of motility related genes was lower compared to carbon ion irradiation. For further confirmation by RT-qPCR, we focused on six genes of which the expression was at least four times decreased after 2.0 Gy of carbon ion irradiation (*CCDC88A*, *ROCK1*, *NEXN*, *FN1*, *MYH10* and *MYH9*). These RT-qPCR results validated the observed changes as seen in the microarray analysis.

### Fibronectin 1

Downregulation of *FN1* gene expression was about four times stronger after 2.0 Gy carbon ion irradiation compared to 2.0 Gy X-rays, whereas irradiation with a dose of 0.5 Gy of either beam quality did not induce a significant change. *FN1* codes for a glycoprotein and is involved in the integrin signaling pathway (52,53), thereby playing an important role in tissue organization, cell adhesion and cell migration. In addition, fibronectin has been reported to be involved in tumor metastasis (54–56). Influence of low-LET irradiation on fibronectin expression has been reported previously by Andarawewa *et al* (57). They found that human mammary epithelial cells exposed to a combination of 2 Gy X-ray and TGF-β underwent a morphological shift from an epithelial to a mesenchymal phenotype. This shift included a higher expression of fibronectin and led to increased cell motility and elevated invasion potential of epithelial cells *in vitro*. They suggested that irradiation of these preneoplastic cells with moderate doses prime them to undergo epithelial to mesenchymal transition (EMT), which could accelerate cancer progression. Additionally, Yang *et al* (58) irradiated a radio-sensitive and a radioresistant non-small cell lung carcinoma cell line with 2 Gy γ-irradiation and analyzed amongst others the expression of *FN1*. Their results indicated that 4 h after irradiation gene expression of *FN1* was not changed in either cell lines. On the other hand, Hei *et al* (59) found that exposure of immortalized human lung and breast epithelial cells to 0.6 Gy high LET α-particles (LET=150 keV/*μ*m) induced changes in fibronectin gene expression. Although, northern blot analysis could not confirm these results.

So far, different studies observed different effects of radiation on *FN1* expression. Whether downregulation of *FN1* in irradiated PC3 cells will result in changes in cellular behavior needs further investigation.

### Actin-binding proteins

The other five motility genes (*ROCK1*, *CCDC88A*, *NEXN*, *MYH9* and *MYH10*), which we selected for further investigation, code for actin-binding proteins. It is well known that remodeling of the actin-myosin skeleton by activation of the serine/threonine kinase Akt can lead to changes in migration, invasion and metastasis (60).

The *ROCK1* gene codes for the Rho-associated, coiled coil containing protein kinase 1, which is a downstream effector of the Rho-pathway, that seems to play a role in tumor metastasis and invasion by influencing cell adhesion and migration capacity (61,62). Lin *et al* (62) transfected PC3 cells with mir-146a, a microRNA found to be downregulated in hormone-refractory prostate tumors, which targets *ROCK1*. Suppression of *ROCK1* in these prostate cancer cells reduced cell proliferation, invasion capacity and their adhesion potential to bone marrow endothelial cells *in vitro*. Therefore, it was suggested that *ROCK1* suppression could aid in reducing the metastatic potential of prostate cancer cells. Furthermore, the effect of radiation on *ROCK1* expression has been previously studied. Zhai *et al* (63) irradiated three glioblastoma cell lines with different doses (ranging from 0 to 8 Gy) of photon radiation and observed a dose-dependent, enhanced invasive potential using *in vitro* assays. They hypothesized that radiation-induced activation of the PI-3K pathway affected Rho/ ROCK1 signaling. They found that ROCK inhibition significantly reduced radiation-induced cellular invasion, whereas inhibition of ROCK without radiation exposure had no effect on the invasion potential. In a study of Fujita *et al* (31), pancreatic cancer cell lines were irradiated with either carbon ions or X-rays. They observed that cancer cells were able to switch from a mesenchymal mode of motility to a protease-independent mechanism of invasion which was based on actomyosin contractility dependent on ROCK signaling. They found that although carbon ion radiation was capable of decreasing the metastatic potential of these cells, ROCK inhibition was needed to block invasiveness. In the present study, we found that radiation exposure decreased the expression of *ROCK1* in a dose-dependent way in prostate cancer cells. This effect was more pronounced after 2.0 Gy carbon ion irradiation compared to 2.0 Gy X-rays, indicating that the magnitude of downregulation is radiation quality-dependent. Since previous results demonstrated that inhibition of ROCK attenuated cell migration capacity, our findings suggest that carbon ion irradiation could be more efficient in decreasing the migration potential of some cell lines.

One of the functions of ROCK1 is the phosphorylation of the myosin light chains. Together with the myosin heavy chains (MYH), these proteins are responsible for the motor function of the actin-myosin complex, thereby driving various motility-based processes such as cytokinesis, cell rounding and cell migration (64). *MYH9* and *MYH10* genes encode for isoforms of non-muscle myosin II heavy chains (65,66). MYH9 was reported to be involved in the formation of lamellopodia at the leading edge of breast cancer cells (67). Furthermore, reducing the expression of MYH9 in an invasive form of MCF-7 breast cancer cells blocked the invasive potential of these cells (68). Also for *CCDC88A* and *NEXN*, the involvement of these genes in metastases has been described (60,69–71). *CCDC88A*, also known as *GIV* or *Girdin*, codes for coiled-coil domain containing 88A, and was found to be more highly expressed in samples of colorectal cancers with liver metastasis compared to cancers without metastasis (60). Garcia-Marcos *et al* (69) found the full length protein exclusively expressed in highly invasive colon, breast and pancreatic tumors, suggesting the protein to be a useful clinical marker for metastatic potential. Finally, NEXN was reported to be involved in cell motility and adhesion of HeLa cancer cells through the binding link from actin and the plasma membrane (71). To our knowledge no studies specifically focused on the influence of radiation on the expression of genes coding for the actin-binding proteins CCDC88A, NEXN, MYH9 and MYH10. However, Fushimi *et al* (36) irradiated oral squamous cell carcinoma cell lines with X-rays (2, 4 and 8 Gy), carbon ions or neon ions (1, 4 and 7 Gy; LET=78 keV/*μ*m) and performed Ingenuity pathway analysis. They indicated integrin, actin cytoskeleton and PI3K/Akt signaling as affected pathways. The genes found in our study also play a role in these motility regulating pathways. Additionally, Akino *et al* (29) found that 12 h after irradiation of non-small-cell lung (NSLC) cancer cells with 5 Gy carbon ions the expression of the actin-binding protein anillin (ANLN) was downregulated, while irradiation with X-rays increased ANLN expression.

As we mentioned before differences in genes which are significantly expressed after irradiation can be due to use of other time-points taken for the sampling. Therefore, it could be informative to further investigate how the expression of actin-binding proteins in response to different radiation qualities changes during time.

In this study, we found that exposure of prostate cancer cells to different radiation qualities decreased the expression of these five genes coding for actin-binding proteins. Furthermore, enrichment analysis indicated that 1.54% of all significantly regulated genes are involved in actin-binding processes (Fig. 2), and further GSEA analysis indicated many gene sets involved in actin-regulation. In view of the importance of actin-binding and actin remodeling in migration processes, it will be important to investigate whether this downregulation may lead to changes in cellular behavior, thereby influencing the migration potential of the cells. Important to mention is that during radiotherapy most of the cancer cells will be eliminated because of the lethal dose. However, some cells can manage to survive, either because they receive sub-lethal doses and/or because they successfully induce their repair mechanisms. We are aware that in this study, only one time-point was analyzed (8 h) after irradiation with a single dose. Further studies could include more time-points, as well as fractionated irradiation to get a more complete picture of the differences in cell migration after low- and high-LET radiation.

### Clinically relevant biomarkers in prostate cancer

In view of the involvement of these six genes in cancer cell motility we also investigated the importance of our selected genes in prostate cancer prognoses by performing a recurrence-free survival analysis on two independent sets of prostate cancer patients (47,48). *FN1* was the only gene of which high expression was significantly correlated with higher recurrence in both data sets. This gene has been previously identified as a potential biomarker for radiation resistance after genomic analysis of several radioresistant head and neck squamous cell carcinomas (72). Futhermore, Hébrant *et al* (70) observed that not only *FN1* but also *CCDC88A* and *MYH9* were more highly expressed in more aggressive anaplastic thyroid carcinomas compared to papillary thyroid carcinomas. We observed an obvious drop in survival rates in both patient groups with high *FN1* expression although there was a difference in the percentage of recurrence-free survival. However, this may be explained by the fact that we took publicly accessible data which were processed in a different manner by the original authors. We appointed high-intermediate-low gene expression based on different criteria in both sets. Adjusting the criteria slightly could equalize the difference in survival percentage. Performing the same analysis on an even larger study population will give more information on how recurrence-free survival may be correlated to *FN1* gene expression.

Survival analysis was also in agreement for both data sets for *MYH10* expression and *NEXN* expression. *MYH10* expression could not be used to distinguish differences in recurrence between expression groups, while low *NEXN* expression correlated with poor survival rates. On the other hand *CCDC88A*, *MYH9* and *ROCK1* expression predicted a different patient outcome in the two study populations. Low *MYH9* or *ROCK1* expression tended to be correlated with better recurrence-free survival for the patients of Gulzar *et al* (47), while *CCDC88A* did not distinguish survival between low and high gene expression groups. However, for the patients of Taylor *et al* (48) low *CCDC88A* or low *ROCK1* expression predicted a poor prognosis for the patients and *MYH9* expression could not distinguish differences in survival based on low or high expression. We only found one other study investigating the effect of ROCK1 protein expression in osteosarcoma samples on patient survival (73). Liu *et al* correlated high ROCK1 presence with lower overall survival rates (73). Within our analysis both patient groups gave contrary results. Also other authors pointed out the difficulties of finding reliable prognostic markers within different sets of prostate cancer patients (47,48). We conclude that these five actin-binding genes may not be suitable biomarkers for prostate cancer prognosis, however, our finding that they are downregulated after radiation exposure in PC3 cells may lead to changes in cell behavior after irradiation.

Although we focused on these five genes in the context of cell migration, it should be noted that actin remodeling is also a very important process during cell cycle progression and mitosis as well. Therefore, it is possible that the observed changes are part of, or a side effect of, alterations in cell cycle due to radiation exposure rather than an actual change in migration potential of the cell. The present study should thus be extended with *in vitro* and *in vivo* assays to verify whether the observed changes are permanent, thereby leading to a change in cellular phenotype or returning to baseline without major consequences for the cell. These assays could bring more insight into the clinical radiation effects induced in patients.

The main goal of this study was to analyze the effects of different types of radiation (carbon ions vs. X-rays) on the gene expression of prostate cancer cells. It is well known that changes in gene expression play a key role in cancer progression (18,19).

Dysregulation of signaling pathways in surviving cells after radiotherapy, such as those associated with cell migration and motility, can determine the fate of the tumor and consequently the fate of the patient. Since most prostate cancer-related deaths are due to metastases (9–11) it is of pivotal importance to understand how therapeutic intervention can influence metastatic development of irradiated cancer cells. Furthermore, cases of radiation-induced metastasis in prostate cancer patients have been reported (74–77). Since clinical trials, using optimized settings for carbon ion treatment, have shown high survival rates and good local control (8,12–15), carbon ion irradiation has been approved as a valid treatment for prostate cancers (8). However, in view of this new treatment option it is of high importance to further investigate changes in cancer cells that survived the treatment in the context of potential long-term effects.

We found that, under the conditions investigated, carbon ion irradiation induced more pronounced changes in PC3 cells in terms of number of genes and magnitude of changes compared to X-rays. We more specifically focused on genes involved in cell motility and found that these genes were generally downregulated after irradiation, an effect which was more pronounced after carbon ion irradiation compared to X-rays. This irradiation-induced downregulation may suggest a suppressed migration potential of PC3 cells, especially after carbon ion irradiation. Further research is needed to investigate whether PC3 cells show a decrease in migration potential after exposure to different radiation qualities. In this regard, functional assays on cell motility and invasion, such as the scratch healing assay and Boyden chamber assay can help in determining whether the observed changes also influence the cellular behavior after irradiation. Understanding how different radiation qualities affect the migration potential of prostate cancer cells is important for improving the clinical outcome of cancer radiation therapy.

## Figures and Tables

**Table I.** Cell motility gene set.

Name gene set	Website	No. of genes in set	No. of genes found in data set	No. of significantly regulated genes (after 2.0 Gy C-ion)
GO0048870 - Cell motility	Gene Ontology	715	746	50

**Table II.** The Applied Biosystems assays.

Gene symbol	Gene name	Assay ID	Ref seq	Exon boundary	Measured efficiency
*FN1*	Fibronectin 1	Hs01549967_m1	NM_002026.2	3–4	1.98
*MYH9*	Myosin; heavy chain 9; non-muscle	Hs01066369_m1	NM_002473.4	23–24	1.96
*NEXN*	Nexilin	Hs00332124_m1	NM_144573.3	10–11	1.91
*CCDC88A*	Coiled-coil domain containing 88A	Hs01559766_m1	NM_001135597.1	18–19	1.98
*ROCK1*	Rho-associated; coiled-coil containing protein kinase 1	Hs01127714_mH	NM_005406.2	4–5	1.99
*MYH10*	Myosin; heavy chain 10; non-muscle	Hs00992050_m1	NM_005964.1	21–22	2.01
*B2M*	β-2-microglobulin	Hs00984230_m1	NM_004048.2	3–4	2.05

**Table III.** Significantly expressed genes: FC ≤-2 or FC ≥2 and FDR ≤0.05.

Radiation	Total no. of genes	Downregulated	Upregulated	No. of unidentified genes
0.5 Gy C-ion	0			
2.0 Gy C-ion	1663	1145	518	248
0.5 Gy X-ray	0			
2.0 Gy X-ray	396	312	84	39

FDR, false discovery rate; FC, fold-change.

**Table IV.** Significantly regulated motility genes: FC ≤-2 or FC ≥2 and FDR ≤0.05.

Radiation	Total no.of genes	Downregulated genes	Upregulated genes
0.5 Gy C-ion	0		
2.0 Gy C-ion	50	46	4
		*ABR, APBB2, APC, ARID5B, BBS1, CCDC88A, CD2AP, DPP4, EGFR, FN1, FYN, GAB2, HOXA5, HOXA7, HOXB9, ICAM1, JMY, L1CAM, LAMC1, MIA3, MYH10, MYH9, NAV1, NDE1, NEXN, NF1, NR4A2, NRP2, PIK3CA, PIK3R1, PPARD, PRKCA, PROS1, PROS1, PTPRM, RNF20, ROBO1, ROCK1, SOS1, SPAG9, SRGAP1, SYNE2, TOP2B, VCAN, VHL, WWC1*	*CXCL3, MIF, PRSS3, S100A2*
0.5 Gy X-ray	0		
2.0 Gy X-ray	15	15	0
		*CCDC88A, FN1, GAB2, JMY, L1CAM, LAMC1, MIA3, MYH10, NEXN, PROS1, PROS1, ROBO1, ROCK1, SYNE2, TOP2B*	

FDR, false discovery rate; FC, fold-change.

**Table V.** Microarray data of motility genes selected for RT-qPCR confirmation.

Gene symbol	FC 0.5 Gy C-ion	FC 2.0 Gy C-ion^a^	FC 0.5 Gy X-ray	FC 2.0 Gy X-ray^a^
*FN1*	−1.27	−8.73	−1.03	−2.06
*CCDC88A*	−2.27	−13.87	−1.32	−4.21
*ROCK1*	−1.57	−11.86	−1.09	−3.27
*MYH9*	−1.18	−4.46	−1.02	−1.73
*NEXN*	−1.49	−8.36	−1.16	−2.49
*MYH10*	−1.36	−4.81	−1.15	−2.21

a Statistically significant: FDR P≤0.05. FDR, false discovery rate; FC, fold-change.
